# Development and Pilot Test of a Virtual Reality Respiratory Biofeedback Approach

**DOI:** 10.1007/s10484-020-09468-x

**Published:** 2020-05-02

**Authors:** Johannes Blum, Christoph Rockstroh, Anja S. Göritz

**Affiliations:** Department of Occupational and Consumer Psychology, Albert-Ludwigs-Universität Freiburg, Engelbergerstr. 41, 79106 Freiburg, Germany

**Keywords:** Virtual reality, Respiratory biofeedback, Diaphragmatic breathing, Abdominal breathing, Respiratory sinus arrhythmia, Biofeedback

## Abstract

Breathing exercises with biofeedback have benefits over breathing exercises without biofeedback. However, the traditional measurement of respiratory signals that is required as part of feeding back the breath incurs high cost and effort. We propose a novel virtual reality (VR) based approach to respiratory biofeedback that utilizes the positionally tracked hand controllers integrated into modern VR systems to capture and feedback the respiration-induced abdominal movements. In a randomized controlled laboratory study, we investigated the feasibility and efficacy of the developed biofeedback algorithm. In total, 72 participants performed a short breathing exercise in VR with or without respiratory biofeedback. The feedback integration resulted in a satisfactory user experience, a heightened breath awareness, a greater focus on slow diaphragmatic breathing and an increased respiratory sinus arrhythmia. This evidences that the novel biofeedback approach is low-cost, unobtrusive, usable and effective in increasing breath awareness and promoting slow diaphragmatic breathing in the context of VR-based breathing exercises. Future studies need to investigate the broader applicability and long-term effects.

## Psychophysiological Benefits of Conscious Breathing

In our fast-paced society, stress-related symptoms and disorders are prevalent (American Psychological Association 2018; Jarczok et al. 2013). Breathing exercises are a practicable, efficient and evidence-based approach to reduce stress-related symptoms and improve psychophysiological health (e.g., Hopper et al. 2019; Perciavalle et al. 2017). Fostering breath awareness and a slow diaphragmatic breathing style is beneficial from a psychological as well as physiological point of view. In the psychological domain, the breath can act as an anchor for focused attention. This is a common feature in a variety of yoga and meditation practices as well as in panic or anxiety interventions. Within those contexts, focused breathing exercises have been found to reduce mind wandering (e.g., Burg and Michalak 2011; Xu et al. 2017) as well as anxiety and negative affect (e.g., Jerath et al. 2015; Ma et al. 2017; Sharma & Haider, 2013). Moreover, focused breathing can foster a positive mood and relaxation (e.g., Blase and van Waning 2019; Busch et al. 2012; Hopper et al. 2019; Perciavalle et al. 2017; Szabo & Kocsis, 2017).

From a physiological point of view, conscious breathing helps maintain or regain balance between the sympathetic and parasympathetic branch of the autonomic nervous system by stimulating the parasympathetic system. The breath is linked to the cardiovascular system through what is referred to as *respiratory sinus arrhythmia* (RSA; Hayano et al. 1996). Controlled by regulatory mechanisms sensitive to changes in blood chemistry and intrathoracic pressure (cf. Porges 2007), inhaling temporarily inhibits vagal tone (the main contributor to the parasympathetic branch), which in turn increases heart rate and decreases blood pressure. By contrast, exhaling temporarily increases vagal tone, which decreases heart rate and increases blood pressure. Breathing at a low frequency (around 0.1 Hz, i.e., six breath cycles per minute; cf. Lehrer and Gevirtz 2014) pronounces the modulation of the parasympathetic nervous system, maximizes the RSA, and thus exercises the autonomic reflexes to make them work more efficiently (Vaschillo et al. 2006; Yasuma and Hayano 2004). Thereby, slow diaphragmatic breathing can help exercise and rebalance the autonomic nervous system.

The psychological and the physiological effects of slow breathing affect each other in a double feedback loop. Modulation and increased activation of the parasympathetic nervous system through slow regular breathing calms the body and leads to mental relaxation (see research on heart rate variability biofeedback, e.g., Gevirtz, 2013; Goessl et al. 2017; Kennedy and Parker 2018; Wheat and Larkin 2010). A calm and relaxed mind, in turn, facilitates a clear and conscious focus on the breath and thus helps breathe more regularly, which is a prerequisite to an increased RSA.

## Respiratory Biofeedback

Although focused breathing is a cost-free and low-effort way to boost psychophysiological health, continuous practice is not straightforward. Whether with breathing meditation or focused breathing exercises, people often struggle to sustain their motivation, fail to keep their focus on the breath, or lack sufficient self-awareness (Pisa et al. 2017; Soyka et al. 2016). All of this may hinder continuous practice or may lead to frustration. Struggling with an adequate performance due to boredom or uncertainty as to the correct behavior may keep many people from establishing the desired routine.

To enhance engagement in breathing exercises and to provide more guidance for continuous practice, one may make the user directly aware of their breathing state through respiratory biofeedback. Respiratory signals (inhalation, exhalation) can be detected via different sensors and fed back to the user in real time, typically in the form of visual or auditory stimuli (for a review, see Giggins et al. 2013; Yu et al. 2018). There are a number of different approaches to quantify the user’s respiration depending on the targeted breathing style and context, such as measuring the amount or temperature of airflow from mouth or nostrils, capturing thoracic or abdominal movement, or detecting subtle breath-induced noise. In case of using biofeedback to raise the breath awareness in a relaxation-focused breathing exercise, a widespread goal is to train diaphragmatic breathing (Giggins et al. 2013), which is considered to be physiologically superior to thoracic breathing (cf. Ma et al. 2017). With diaphragmatic breathing, sensors located on the abdomen (e.g., strain gauges, accelerometers, linear potentiometers) pick up respiration-induced abdominal movements, which are then used to infer respiration. The detected signals (inhalation, exhalation) can be fed back to the user directly (e.g., Tinga et al. 2019; van Rooij et al. 2016; Vidyarthi and Riecke 2013) or in the form of pre-processed, aggregated parameters such as respiratory frequency or depth (e.g., Bhandari et al. 2015; Harris et al. 2014; Morarend et al. 2011; Parnandi et al. 2013). The feedback can serve several goals. First, it can raise awareness towards the breath because one’s own breathing actions and changes are immediately and easily observable. Second, respiratory feedback can be used to evaluate the current breathing style, inform the user about it, and, if necessary, promote the desired breathing style (e.g., slow and even respiration). Third, the feedback is often designed in a pleasing way to be rewarding and thus allows for reinforcement learning (Gaume et al. 2016; Sherlin et al. 2011).

The success of a biofeedback exercise to raise awareness partly depends on the visual appeal and rewarding characteristic of the biofeedback system and stimuli. To optimize the engagement with and the level of control over biofeedback exercises, a growing number of studies have investigated the feasibility and effectiveness of using head-mounted virtual reality (VR) to deliver biofeedback (e.g., Blum et al. 2019; Gromala et al. 2015; Rockstroh et al. 2020; Tinga et al. 2019; van Rooij et al. 2016; Weerdmeester et al. 2017). Due to the recent technological advances in the field, VR has become an accessible and affordable technology. High levels of immersion and sense of presence (Cummings and Bailenson 2016; Makransky et al. 2019) make VR a promising tool for subjective, experience driven behavioral treatments while the virtual environment offers a maximum of control for training and research purposes.

In the context of respiratory biofeedback exercises, VR offers two main benefits. First, high immersion through stereoscopic, six degrees-of-freedom, head-mounted VR can help create and implement vivid and visually appealing feedback stimuli, which have been shown to increase motivation and engagement (Rockstroh et al. 2019; Soyka et al. 2016; van Rooij et al. 2016). It can be argued that the more attractive a stimulus, the more salient it is to the user who then develops a greater engagement and motivation to establish a training routine. This argument is also loosely based on the principle of *gamification* (Robson et al. 2015), an approach in which motivational elements of game design are utilized to keep up user motivation in game-like interventions that have an educational or therapeutic goal instead of mere entertainment (serious games). Second, besides making the exercise more interesting and engaging through greater visual fidelity of the key elements, VR offers an easily controllable and customizable setting for the exercise. By being able to control not only the feedback itself but also the surrounding environment, unwanted distractions as well as boring or unpleasant rooms or scenery can be diminished. The environment can be designed to support relaxation on the one hand and draw effortless, involuntary attention on the other hand by presenting calm and appealing surroundings (e.g., virtual beach: Blum et al. 2019; virtual forest: Gromala et al. 2015; virtual underwater world: van Rooij et al. 2016).

## Proposal of an Integrated Respiratory Biofeedback Approach in VR

Despite the feasibility of respiratory biofeedback to enhance breath awareness, a broad application is held back by practical and economic constraints, which have restricted respiratory biofeedback to clinical or laboratory settings so far. The customary measurement approaches require a significant amount of effort and expertise in administering biofeedback. Costly sensors, amplifiers, interfaces, as well as software setup and integration need to be considered. Moreover, the measurement is often obtrusive (e.g., facial mask for airflow detection, nostril sensors) and might intimidate the user, which hinders the relaxation purposes of the exercise. In the context of VR-based biofeedback applications, these concerns are even more pronounced due to the additional complexity of integrating the systems.

In the present paper, we present a VR-based respiratory biofeedback paradigm that aims to deliver the benefits associated with respiratory biofeedback while avoiding the common critique by providing an integrated, cost-effective, easy to use and fairly unobtrusive approach. We developed an algorithm that makes use of the hand controller of a VR headset to capture the respiration-induced abdominal movements in real time without the need for additional devices or systems. Contemporary VR headsets come with a set of hand controllers that are positionally tracked in 3D space. At any time, the position of the controller can be determined with great precision. If placed on the abdomen, the relative positional changes of the controller over time can be used to approximate the respiration-induced movements of the abdomen. Expansion of the abdomen (inhalation) pushes the controller slightly forward relative to the user. Contraction of the abdomen (exhalation) pulls the controller slightly backwards relative to the user. The subtle forward and backward controller movements can be processed with the following steps:

1. Determine the target vector of the controller, that is, the axis orthogonal to the abdomen. In VR, the user can move and rotate freely with six degrees of freedom (3 positional axes, 3 rotational axes). The same applies to the controller. Independent of the user’s and controller’s current position or rotation, any controller movement along the axis orthogonal to the user’s abdomen indicates diaphragmatic breathing. Any movement along a non-target vector as well as any controller rotation indicate controller movement artifacts unrelated to diaphragmatic breathing.
2. For each time frame (depending on the refresh rate of the VR headset ca. every 11 to 14 ms), determine the positional delta to the previous frame along the target vector. Accumulate (sum up) the delta values over time to retrieve an estimate of the absolute controller position along the target vector for each frame; record the summed delta value at each frame to generate a summed-delta-series over time.
3. Compute a short moving-average for smoothing (e.g., 10 frames) and a long moving-average for trend analysis (e.g., 90 frames) of the summed-delta-series and compare the two values at each frame. The resulting trend value allows for a real time classification of the current respiratory status within each frame: A positive value indicates forward abdominal movement (i.e., inhalation) and a negative value indicates backward abdominal movement (i.e., exhalation); a small threshold area around zero can be used to control for noise.
4. To control for movement artifacts unrelated to respiration, check for controller movement along a non-target vector (unrelated positional delta) and controller rotation (rotational delta) within each frame. If any of the two occur, proper diaphragmatic breathing is unlikely. For those instances, the respiratory status is defined as movement artifact.

The current respiratory status can be fed back to the user in real time. The proposed measurement paradigm has advantages over existing implementations. Most obviously, it does not depend on an external device or sensorics system that would need to be integrated with the VR system. Additionally, it can work with different VR devices, manufacturers or tracking technologies (outside-in as well as inside-out tracking), as long as the VR system implements precise positional tracking of the controllers. Moreover, the controller that acts as a sensor can be held in place manually or with the help of an elastic belt. Either way, it provides a ready to use respiration sensor at no additional cost and presumably without any discomfort.

We conducted an empirical laboratory study to empirically test the functionality of the developed approach including the algorithm in a VR-based focused breathing exercise. The study was designed to evaluate (1) the user experience and feasibility of the developed approach and (2) its ability to foster focused diaphragmatic breathing. Alongside a group of participants who tested the developed respiratory biofeedback approach, we ran a comparable control condition without the inclusion of biofeedback.

## Methods

### Participants and Design

Participants were recruited via social media as well as an online database. While there was no monetary reward for participation, undergraduates could receive course credit. In total, 72 healthy undergraduate participants (56 women) took part in the experiment. The age ranged from 18 to 49 with an average of 21.6 years (*SD* = 4.3). None of the participants had any prior experience with respiratory biofeedback in VR.

The study was conducted as a randomized, controlled, between-subjects, laboratory experiment with written informed consent. The participants were randomly assigned to one out of two conditions, a focused breathing exercise in VR (control group, *n* = 36), or a respiratory biofeedback in VR (feedback group, *n* = 36). We assessed user experience (post-exercise), subjective breath awareness (post-exercise), respiratory induced abdominal movements (during the exercise) and heart rate variability (during the exercise) as dependent variables. In the end, participants were debriefed. The entire experiment including the randomization was PC-based and thus double-blind.

## VR Breathing Exercises

**Fig. 1:**
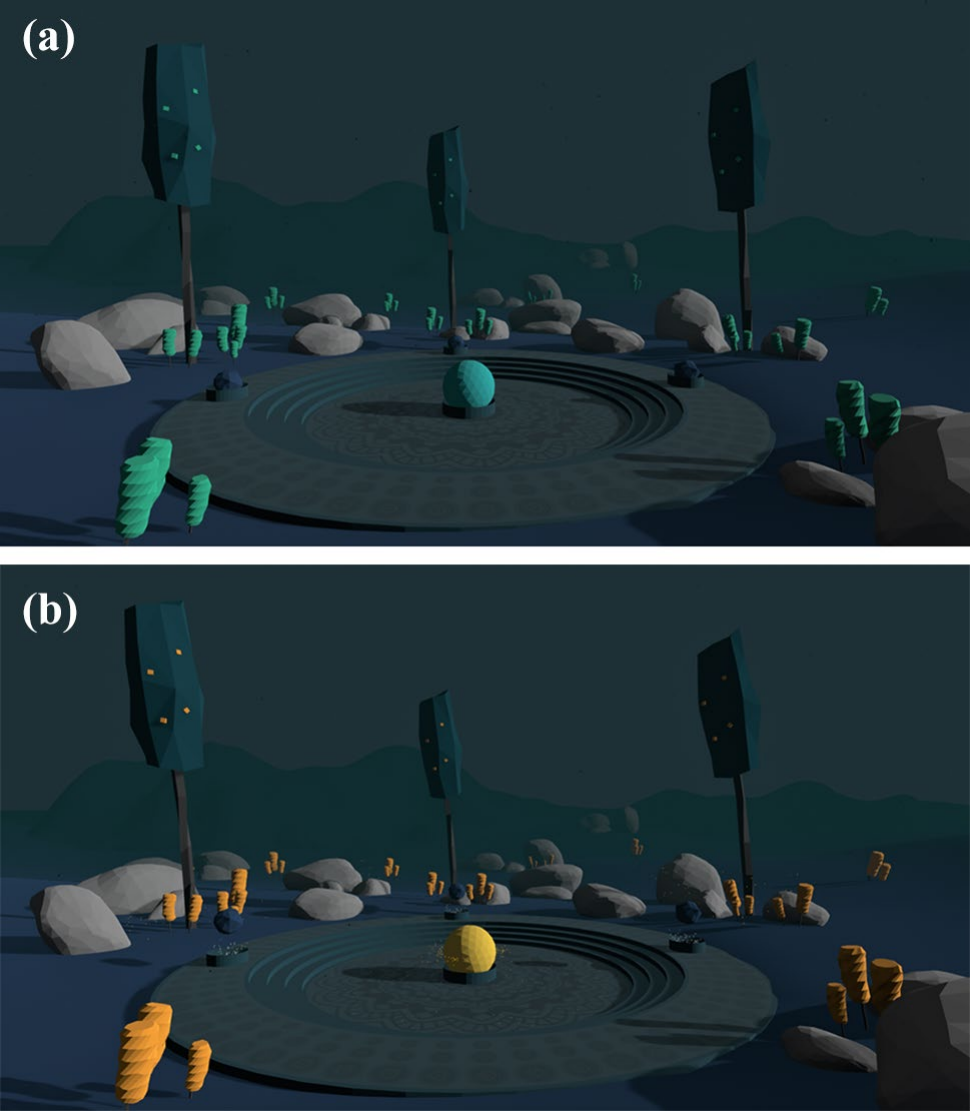
Screenshot of the virtual environment in its default state (**a**) and while exhaling (**b**)

In both experimental conditions, participants experienced the same virtual environment (Fig. 1), presented via an Oculus Rift CV1 head-mounted display. The environment depicted a stylized natural landscape with hills, rocks, flowers and gently swaying trees. We deliberately chose an abstract presentation in terms of colors and shapes to not trigger explicit associations in the participants. Some of the elements in the scene (flowers, parts of the trees, some rocks) were designed to be able to change their color. In the control group, these color changes happened independently of the participants’ respiration by chance. In the feedback group, these elements served as respiratory feedback and changed their color whenever the participant was exhaling. The color change lasted as long as the respective exhalation.

Participants in both groups were instructed to focus on their breath and practice slow diaphragmatic breathing. We emphasized that participants should perform diaphragmatic breathing instead of thoracic breathing. In the feedback group, participants were also informed about the respiratory feedback elements. The whole breathing exercise lasted for seven minutes. In both groups, participants wore a belt around the abdomen that held the VR-controller in place. They were informed that the belt measured their diaphragmatic breathing.

## Instruments

User experience was assessed via four subscales of the User Experience Questionnaire (UEQ; Laugwitz et al. 2008) that measure attractiveness (6 items), perspicuity (4 items), novelty (4 items) and stimulation (4 items) on 7-point semantic differentials.

Focus on the breath was measured via a single item. Participants were asked to rate how much they had succeeded in focusing on their breath on a visual analogue scale (0 = *not at all* to 1 = *very much*).

A VR-controller in conjunction with the algorithm described in the introduction was used to derive abdominal movement parameters. Throughout the exercise, we recorded the current respiratory status (inhalation movement, exhalation movement, movement artifacts or no movement) as outlined in the algorithm description. Based on that, we computed three different types of respiration parameters. First, we computed the relative share (percentage) of the total exercise duration of all inhalation movements, all exhalation movements, all movement artifacts and all phases without any controller movement (i.e., no abdominal movement). Second, we computed the mean durations (sec) of inhalation movements and exhalation movements, that is, the average time periods for expansion or contraction of the diaphragm. This indicates the average duration of a single inhale or exhale respectively. Third, although our approach does not allow for an exact measurement of participants’ respiratory rate (because no thoracic movement data was recorded), we approximated respiratory rate from the raw abdominal movement trend values (cf. Step 3 of the above-described algorithm). Performing a *Fast-Fourier* transform (Welch’s periodogram with a sampling frequency of 90 Hz; using the *signal* function of the *SciPy* python package by Virtanen et al. 2020) on these trend values yielded power spectral density estimates regarding the relative contribution of different frequencies to generate the input signal (here: abdominal movement). Respiratory rate (in Hz) was defined as the peak of the power spectrum distribution within the reasonable bounds of 0.05 to 0.5 Hz.

To assess participants’ RSA during the exercise, intervals between adjacent heartbeats (interbeat intervals in milliseconds) were obtained wirelessly via a Polar H10 chest strap. In line with recommendations in heart rate variability research (Laborde et al. 2017; Task Force of The European Society of Cardiology and The North American Society of Pacing and Electrophysiology 1996) we analyzed the most prominent and best-understood parameters: root mean square of successive differences (RMSSD), low frequency band power (LF) and high frequency band power (HF). Using the *hrvanalysis* python package (Champseix 2018), frequency domain indices were computed with the Welch’s method (sampling frequency: 4 Hz; VLF band: 0.003 to 0.04 Hz; LF band: 0.04 to 0.15 Hz; HF band: 0.15 to 0.4 Hz). Interbeat intervals of greater than 1,800 ms or lower than 350 ms were treated as outliers and replaced using linear interpolation.

## Results

### User Experience

The internal consistencies of the four scales of the UEQ ranged from Cronbach’s ɑ = 0.63 (perspicuity) to Cronbach’s ɑ = 0.91 (attractiveness). As depicted in Table 1, overall mean values were high across all subscales in both conditions (all *M* ≥ 5.18). While there are no norm values for the UEQ, the fact that all items are 7-point semantic differentials suggests that mean values above 4 indicate a satisfying user experience. To test for differences in the user experience between the two conditions, we conducted a separate one-way ANOVA for each UEQ subscale. There were no between-subjects differences on any of the UEQ subscales (all *p* ≥ .244), which means that the addition of biofeedback in the feedback group did not significantly alter the user experience.

**Table 1:** Descriptive statistics of the UEQ

UEQ subscale	Control group (*n* = 36) *M* (*SD*)	Feedback group (*n* = 36) *M* (*SD*)
Attractiveness (6 items) Perspicuity (4 items) Novelty (4 items) Stimulation (4 items)	6.25 (0.76) 5.55 (0.91) 5.91 (0.82) 5.28 (1.04)	6.04 (0.77) 5.52 (0.81) 5.69 (0.78) 5.18 (0.86)

*UEQ *User Experience Questionnaire (Laugwitz et al. 2008); all scales comprise 7-point semantic differentials

## Focus on the Breath

**Fig. 2:**
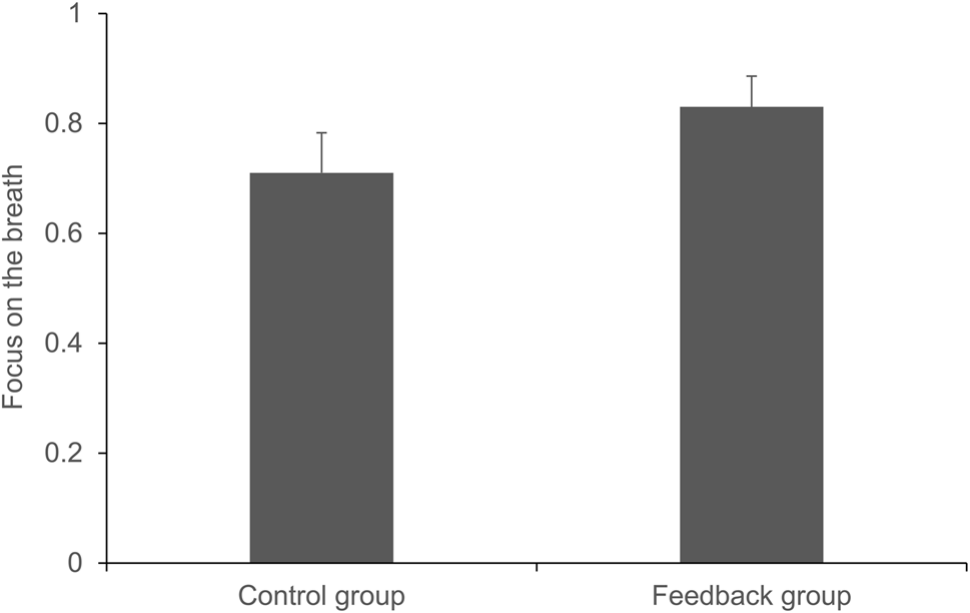
Mean subjective focus on the breath by condition. Error bars represent 95% confidence intervals (CI)

To test for differences in participants’ subjective focus on the breath between the two conditions (Fig. 2), we conducted a one-way ANOVA. There was an effect of the condition on the focus on the breath, *F*(1, 70) = 6.859, *p* = .011, η_p_^2^ = 0.089, with more focus in the feedback group (*M* = 0.83, *SD* = 0.17) compared to the control group (*M* = 0.71, *SD* = 0.22).

## Abdominal Movement

**Fig. 3:**
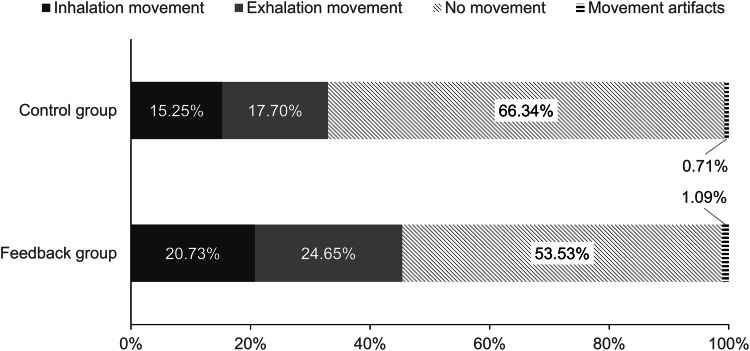
Relative duration of inhalation movement, exhalation movement, movement artifacts and no movement by condition

To explore the effects of the feedback on the amount of using the diaphragm while breathing (Fig. 3), we conducted separate one-way ANOVAs for relative share of inhalation movements (in %), exhalation movements (in %) and movement artifacts (in %). There was no between-groups effect on the relative share of movement artifacts, *F*(1, 70) = 1.287, *p* = .260. In other words, the relative share was comparable between the feedback group (*M* = 1.09, *SD* = 1.49) and the control group (*M* = 0.71, *SD* = 1.35). The ANOVA on the relative share of inhalation movements revealed an effect of the condition, *F*(1, 70) = 8.504, *p* = .005, η_p_^2^ = 0.108, with greater relative share in the feedback group (*M* = 20.73, *SD* = 7.00) compared to the control group (*M* = 15.25, *SD* = 8.84). The ANOVA on the relative share of exhalation movements revealed an effect of the condition, *F*(1, 70) = 9.494, *p* = .003, η_p_^2^ = 0.119, with greater relative share in the feedback group (*M* = 24.65, *SD* = 7.96) compared to the control group (*M* = 17.70, *SD* = 10.94).

**Fig. 4:**
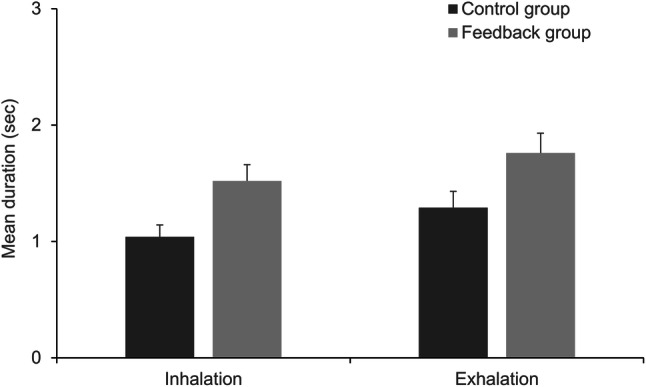
Mean duration (sec) of inhalation and exhalation movements by condition. Error bars represent 95% CI

Furthermore, we conducted two separate one-way ANOVAs on the mean duration of inhalation movements and exhalation movements (Fig. 4). The ANOVA on mean inhalation duration revealed an effect of the condition, *F*(1, 70) = 28.847, *p* < .001, η_p_^2^ = 0.292, with longer average inhales in the feedback group (*M* = 1.52, *SD* = 0.44) compared to the control group (*M* = 1.04, *SD* = 0.31). The ANOVA on mean exhalation duration revealed an effect of the condition, *F*(1, 70) = 18.756, *p* < .001, η_p_^2^ = 0.211, with longer average exhales in the feedback group (*M* = 1.76, *SD* = 0.49) compared to the control group (*M* = 1.29, *SD* = 0.42).

**Fig. 5:**
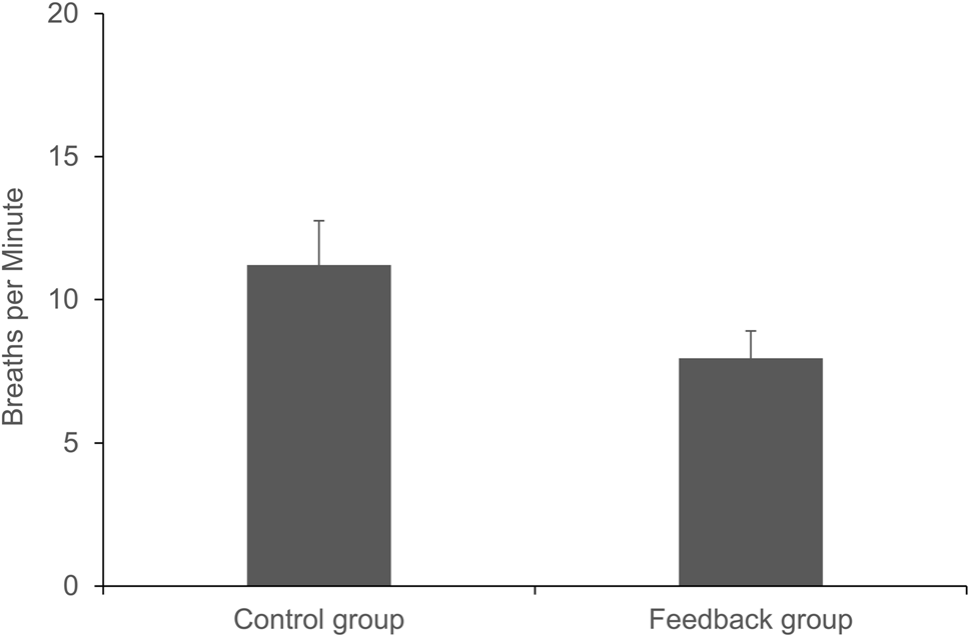
Approximated respiratory rate (breaths per minute) by condition. Error bars represent 95% CI

Additionally, we conducted a one-way ANOVA on the approximated respiratory rate (Fig. 5). The ANOVA revealed an effect of the condition, *F*(1, 70) = 13.168, *p* = .001, η_p_^2^ = 0.158, with a lower respiratory rate (i.e., slower breathing) in the feedback group (*M* = 0.133 Hz [7.98 breaths per minute], *SD* = 0.047 Hz [2.82 breaths per minute]) compared to the control group (*M* = 0.187 Hz [11.22 breaths per minute], *SD* = 0.076 Hz [4.56 breaths per minute]).

## Respiratory Sinus Arrhythmia

**Fig. 6:**
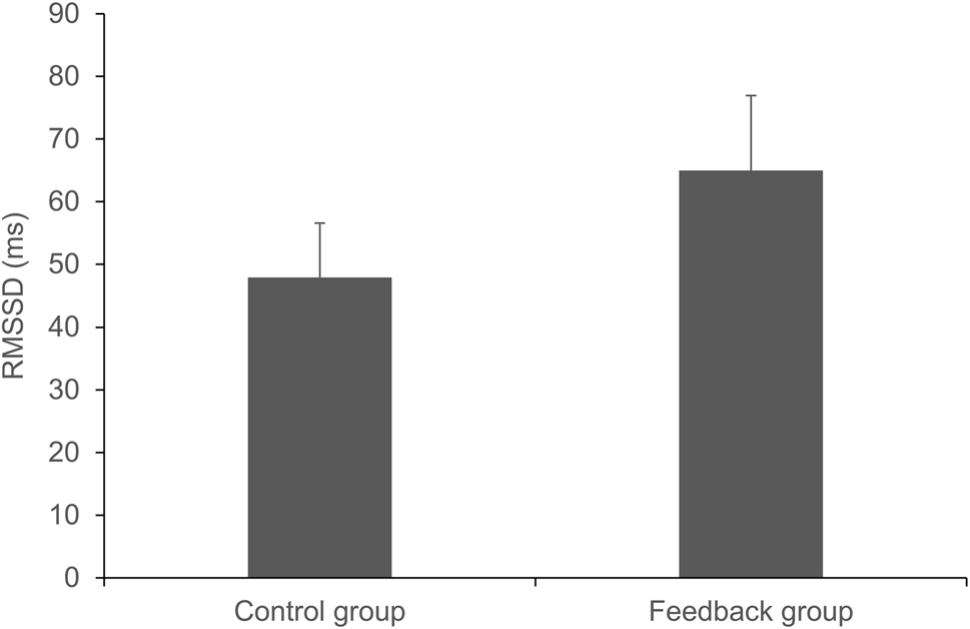
RMSSD (ms) by condition. Error bars represent 95% CI

**Fig. 7:**
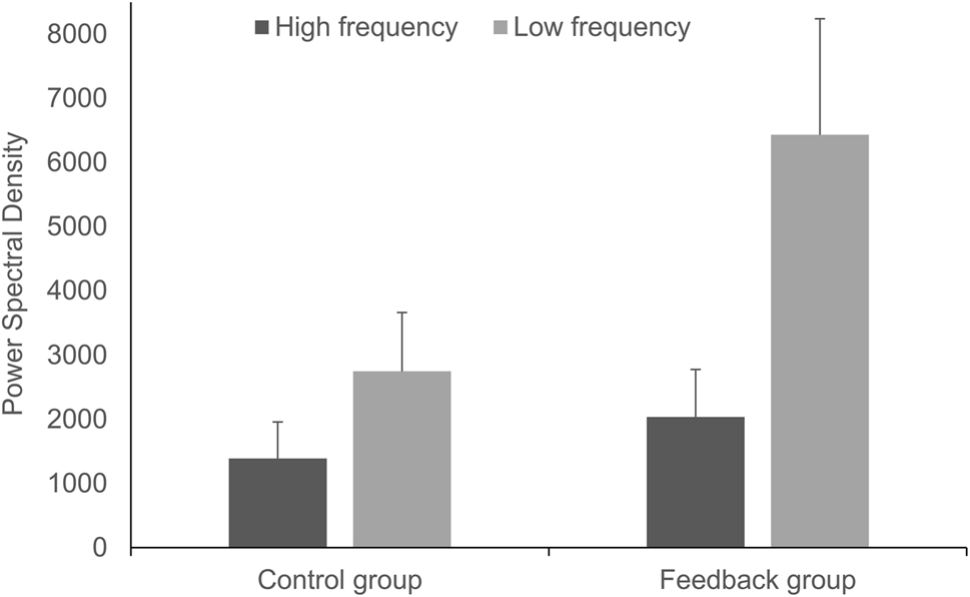
High frequency band power (HF) and low frequency band power (LF) by condition. Error bars represent 95% CI

To investigate the effect of the feedback on participants’ RSA, we computed separate one-way ANOVAs for one time-domain parameter (RMSSD; Fig. 6) and two frequency-domain parameters (LF, HF; Fig. 7) of heart rate variability. The ANOVA on RMSSD revealed an effect of the condition, *F*(1, 70) = 5.498, *p* = .022, η_p_^2^ = 0.073, with higher RMSSD in the feedback group (*M* = 64.96, *SD* = 35.37) compared to the control group (*M* = 47.88, *SD* = 25.68). The ANOVA on LF revealed an effect of the condition, *F*(1, 70) = 13.568, *p* < .001, η_p_^2^ = 0.162, with higher LF in the feedback group (*M* = 6431.58, *SD* = 5349.54) compared to the control group (*M* = 2750.57, *SD* = 2708.01). There was no between-group effect on HF, *F*(1, 70) = 1.992, *p* = .163. In other words, HF was comparable between the feedback group (*M* = 2040.40, *SD* = 2182.39) and the control group (*M* = 1392.46, *SD* = 1680.48).

## Discussion

The present study explored the feasibility and efficacy of a novel VR-based diaphragmatic breathing biofeedback algorithm. We tested the effects of the procedure on the user experience, the subjective and objective focus on diaphragmatic breathing as well as the RSA. The results support the practicability and efficacy of the algorithm to enhance a focus on the breath.

In detail, as regards user experience, there were no differences between the feedback group and the control group on any of the subscales of the UEQ. However, this result does not appear problematic as both groups scored equally and satisfactorily high on all scales. Moreover, given the fact that the VR-based exercise itself was novel in both groups, the fact that the addition of biofeedback in the feedback group did not lower user experience ratings indicates that the developed approach neither adds substantial effort nor diminishes comfort or ease of use. Therefore, we deem the algorithm a feasible and low-cost way of integrating respiratory biofeedback into VR.

As regards the efficacy of the algorithm to enhance the focus on diaphragmatic breathing, the results are positive. Participants reported greater success in keeping their focus on the breath when supported by the biofeedback. The real time measurement and feedback of abdominal movements appears to have helped the participants keep their attentional focus on the breathing.

These self-reports are mirrored by the objective abdominal movement parameters. In both groups, there were only negligible movement artifacts. This indicates that the participants were compliant and did not make strong or sudden movements in VR. The greater self-reported focus on the breath was accompanied by an objective increase in the relative share of abdominal movements (i.e., diaphragm use) with both inhalation and exhalation in the feedback group compared to the control group. It should be noted that phases without any abdominal movement or movement artifacts accounted for more than 50% of the time in both groups. Even with diaphragmatic breathing, phases of breath holding without abdominal movement when switching from inhalation to exhalation and vice versa are common. Nevertheless, it appears implausible that the participants actually held their breath for such a substantial amount of time. Instead, this finding indicates that participants in both groups struggled to perform adequate diaphragmatic breathing, which underlines the requirement for corresponding exercises. Remarkably though, the relative share of abdominal movements (inhalation and exhalation taken together) was higher in the feedback group by more than a third. Taking into account that this happened during a short duration single-session exercise, this is promising.

Moreover, the biofeedback did not only increase the total share of diaphragm use while breathing, it also increased the average duration of respiration-induced abdominal movements, with both inhalation and exhalation. This shows that the increase in diaphragm involvement cannot be ascribed to quicker breathing with less breath holding. Instead, two possible explanations seem likely. First, participants in the feedback group might have made more use of their diaphragm within each individual breath. Second, participants in the feedback group might have breathed at a lower rate. In the present study, the latter cannot be derived with certainty from the abdominal movement data alone. However, it appears highly likely when considering the results of our approximated respiratory rate analyses. Participants in the feedback group breathed slower by more than 3 breaths per minute compared to the control group. Moreover, the average respiratory rate in the feedback group (7.98) was a lot closer to the common resonance breathing frequency of 6 breaths per minute than in the control group (11.22).

 The greater focus on the breath and the seemingly lower breathing rate in the feedback group compared to the control group are also reflected in the respective RSA, as measured via heart rate variability parameters. Participants in the feedback group showed a greater heart rate variability in the time-domain as assessed by RMSSD. RMSSD is a measure of cardiac vagal tone (Laborde et al. 2017), that is, the degree to which the parasympathetic branch of the autonomic nervous system influences the cardiac activity. This supports the assumption that the feedback group focused more on a calm and regular breath and thus exerted greater parasympathetic activity. In the frequency-domain, the results point towards lower respiratory rates. While the slight increase in HF in the feedback group did not reach a conventional level of significance, the LF values were significantly higher in the feedback group. This shows that the feedback group exerted a stronger RSA, specifically in lower frequency ranges. Breathing at a lower rate promotes an increase in LF without affecting or even lowering the HF as the breathing rate approximates the resonance frequency and RSA shifts to the LF range (Laborde et al. 2017; Task Force 1996). This suggests that the stronger use of the diaphragm in the feedback group was likely accompanied by slower and more regular breathing closer to the resonance frequency, independent of the breathing style (diaphragmatic vs. thoracic).

Taken together, our results are promising in a number of ways. They show that the use of a positionally tracked VR controller for breath tracking does not require heightened effort on the part of the participants or pose a threat to usability and comfort of a VR-based breathing exercise. Furthermore, the results indicate that the proposed procedure is a viable and efficacious respiratory biofeedback paradigm. It appears able to enhance participants’ focus on diaphragmatic breathing and helps them breathe more regularly and more slowly.

## Limitations and Future Research

Although this initial evidence is based on a randomized and controlled laboratory study with multiple psychophysiological data sources, some methodological caveats limit the generalizability of the results. These should be considered when interpreting the outcomes, and they call for future studies to corroborate the findings. First, the study was conducted in a controlled environment and comprised undergraduate students. This does not distort the findings per se; moreover, the developed biofeedback paradigm targets healthy adults from all walks of life so that undergraduates are within the target group. Nevertheless, broader contexts and samples need to be tested to establish the procedure’s robustness and clarify the applicability of the results. For instance, future research might investigate the present VR biofeedback procedure in the context of participants with complaints (e.g., anxiety, pain, dyspnea) to explore a potential clinical use for this technique. Second, while the study included a control group, other control conditions would have been plausible. Recent research points towards the urge to include so called placebo feedback groups (i.e., deceiving the participants to believe they engage in biofeedback when the feedback parameters are bogus) when investigating biofeedback (Tinga et al. 2019). Notably, our algorithm does not require any additional devices and, as argued above, does not diminish user experience. Therefore, from a practical stance (and also from an ethical stance), a placebo feedback condition does not appear worthwhile in our case. Nevertheless, from the point of view of fundamental research, the question whether the effects can be ascribed to the actual feedback or how much the effects depend on the participants’ belief to receive feedback, could be subject to future research. On a similar note, a pre-test on the individual ability to perform diaphragmatic breathing would have further controlled the findings. We have no reason to doubt the success of our randomization. Nevertheless, a pre-test would open up the opportunity for double-checking the homogeneity of the groups or even use a matching approach. Third, there are a number of measurement limitations. Subjective breath awareness was assessed via a single self-framed item. The heart rate variability data were collected via a chest strap, which is reliable under most circumstances (Gillinov et al. 2017; Plews et al. 2017), but does not allow for precise artifact correction via visual inspection of the raw ECG signal. Moreover, we put a focus on the practicability, kept the experimental conditions similar to a potential setting for real world application and made sure to avoid overly cumbersome or obtrusive laboratory conditions. Therefore, we decided to forego an additional validated instrument to measure participants’ respiration (facial mask or additional belt sensors). Consequently, the developed approach cannot yet be regarded a validated paradigm for the assessment of respiratory parameters. Instead, the approach and algorithm merely yield abdominal movement parameters, which need to be considered with some caution. Specifically, the analyses regarding the respiratory rate only represent an approximation of the true frequency. The total number of breaths could not be counted due to the lack of thoracic movement data but had to be estimated via a power spectral analysis. This procedure was chosen to provide a result which can easily be compared with existing studies. Fourth, it is unlikely that the current implementation of the approach represents the optimal realization. The algorithm might benefit from fine-tuning of the thresholds and time-windows. Fifth and last, future research should investigate potential long-term effects. It is likely that the beneficial effect of the integrated biofeedback becomes more pronounced over time. Nevertheless, this short-term one-shot experiment cannot clarify this matter. Furthermore, additional post-test measures of diaphragmatic breathing ability could capture whether the practiced breathing technique is retained in unsupported situations. Moreover, it appears worthwhile to develop and investigate different virtual environments as well as different feedback implementations to address the generalizability and broader applicability of the initial evidence.

## Conclusions

This study piloted a novel approach to VR-based respiratory treatments using biofeedback. The respiratory biofeedback algorithm makes use of the positionally tracked hand controllers that are part of modern VR systems to capture and feedback the respiration-induced abdominal movement. The results from the controlled laboratory study show a satisfactory user experience, a heightened breath awareness, a greater focus on slow diaphragmatic breathing and an increased RSA when applying the biofeedback. This initial evidence indicates that the developed paradigm provides a low-cost, unobtrusive, usable and effective way of raising breath awareness and promoting slow regular diaphragmatic breathing in the context of VR-based breathing exercises. Future studies need to investigate the broader applicability alongside the real world and long-term usage.
